# Occurrence prediction of pests and diseases in cotton on the basis of weather factors by long short term memory network

**DOI:** 10.1186/s12859-019-3262-y

**Published:** 2019-12-24

**Authors:** Qingxin Xiao, Weilu Li, Yuanzhong Kai, Peng Chen, Jun Zhang, Bing Wang

**Affiliations:** 1 Institutes of Physical Science and Information Technology, Anhui University, Hefei, 230601 China; 2 School of Life Sciences, Anhui University, Hefei, 230601 China; 3 School of Electrical Engineering and Automation, Anhui University, Hefei, 230601 China; 4 School of Electrical and Information Engineering, Anhui University of Technology, Ma’anshan, 243032 China

**Keywords:** Long short term memory, Weather factors, Association rules analysis, Recurrent neural network, The occurrence of pests and diseases

## Abstract

**Background:**

The occurrence of cotton pests and diseases has always been an important factor affecting the total cotton production. Cotton has a great dependence on environmental factors during its growth, especially climate change. In recent years, machine learning and especially deep learning methods have been widely used in many fields and have achieved good results.

**Methods:**

First, this papaer used the common Aprioro algorithm to find the association rules between weather factors and the occurrence of cotton pests. Then, in this paper, the problem of predicting the occurrence of pests and diseases is formulated as time series prediction, and an LSTM-based method was developed to solve the problem.

**Results:**

The association analysis reveals that moderate temperature, humid air, low wind spreed and rain fall in autumn and winter are more likely to occur cotton pests and diseases. The discovery was then used to predict the occurrence of pests and diseases. Experimental results showed that LSTM performs well on the prediction of occurrence of pests and diseases in cotton fields, and yields the Area Under the Curve (AUC) of 0.97.

**Conclusion:**

Suitable temperature, humidity, low rainfall, low wind speed, suitable sunshine time and low evaporation are more likely to cause cotton pests and diseases. Based on these associations as well as historical weather and pest records, LSTM network is a good predictor for future pest and disease occurrences. Moreover, compared to the traditional machine learning models (i.e., SVM and Random Forest), the LSTM network performs the best.

## Introduction

Cotton is an important economic crop, which occupies a important position in the national economy. However, cotton was always damaged by various pests and diseases during its growth. Perennial pests and diseases can cause about 15–20% economic loss, even up to 50% in some years. Therefore, the control of pests and diseases is crucial to the growth of cotton, which can recover more than 900,000 tons of cotton annually [1]. During cotton growth, many factors can affect the production, of which the most significant one is abnormal climate change. Abnormal climate change can result in the continuous evolution of pests and further make pests adaptive to the environment, which seriously influences the yield and quality and makes it more difficult to control the pests and diseases [2]. Investigating the relationship between pandemic diseases and weather factors is significant for establishing weather-pest forecasting models and improving the long-term prediction of pests and diseases.

Association rule analysis is one of the important methods in data mining, which is a rule-based machine learning method for discovering interesting relations between variables in large databases. It is intended to discover strong rules in databases using some measures of interestingness [3]. Today, association rule mining is applied in many fields including webpage mining [4], intrusion detection [5], continuous production, and bioinformatics [6]. This paper attempted to further verify the correlation between weather factors and pest occurrence through correlation rule analysis, and to explore the potential laws of pest occurrence and weather changes.

Nowadays, the methods of pest control in cotton mainly included pesticide screening, ecological control, biological control [7], etc, where pesticides were always used in cotton fields. They were insecticidally effective and direct in cotton fields, however, most pesticides are highly toxic and often caused serious residual pollution. Subsequently, high efficiency, low degree and environment-friendly new types of pesticide have been tried to develop for the prevention and control. With the rapid development of life sciences, biological control has become a popular direction. Singh et al. evaluated housekeeping genes, and tried to feed/inject sequence-specific double-stranded RNA (dsRNA), which targeted towards downregulation or knockdown of essential genes for causing mortality [8]. However, controversies still existed in the use of gene drive to control pests. The applications for pest control in agriculture will bring important environmental, social and ethical issues [9]. Moreover, many natural works have been developed, such as releasing natural enemies of cotton fields, exploring habits and resources related to habitat control, and attracting natural enemies, which have played an important role in practice. Ecological control seems simple, but there are consequences of species invasion due to the introduction of natural enemies.

With the development of big data and artificial intelligence, more and more researchers have begun to use machine learning methods to solve prediction problems in different fields, and got good results. Bao et al. proposed a model (Network Consistency Projection for Human Microbe-Disease Association prediction, NCPHMDA), which integrated known microbe-disease associations and Gaussian interaction profile kernel similarity for microbes and diseases, and were successfully confirmed by recent published clinical literature partly [10]. Huang et al. proposed a new method based on independent component analysis (ICA) for tumor classification using gene expression data, which showed that the method is efficient and feasible for DNA microarray datasets [11]. At the same time, machine learning-based methods are promising in agriculture and research emphasis is on prevention of pests. Extensive studies have focused on the pest prediction of crops. Ding et al. proposed an automatic detection pipeline on the basis of deep learning technique, which can real-time monitor the occurrence of pests in the field [12]. Zhang et al. developed multiplier feed-forward neutral networks (MLFN), general regression neutral networks (GRNN) and support vector machine (SVM), to predict the occurrence area of dendrolimus superans [13].

Long short term memory (LSTM) is a deep learning model that has attracted much attention in recent years. It was first proposed by Hochreiter and Schmidhuber in 1997 [14], improved by Yann et al. in 2003 [15], and eventually got a wide range of applications. LSTM is a special kind of recurrent neutral network (RNN), which introduces gate mechanism into vanilla RNN to prevent the problem of vanishing gradient or exploding gradient. Li et al. adopted an LSTM-based auto-encoder with generating coherent text units from neural models to preserve and reconstruct multi-sentence paragraphs [16]. Gao et al. presented a mQA model to answer the questions about the content of an image. The model contains four components: an LSTM to extract the question representation, a Convolutional Neural Network (CNN) to extract the visual representation, an LSTM for storing the linguistic context in an answer, and a fusing component to combine the information from the first three components and output the answer [17]. Theis and Bethge introduced a recurrent image model based on multi-dimensional LSTM units for image modeling [18]. Mirshekarian et al. used LSTM units to learn a physiological model of blood glucoses, which has shown outperformed physician predictions [19].

In this paper, we first found that it is more likely to cause cotton pests and diseases during warm, humid, windless, moderately light and other environments in autumn and winter through association rules. It is further confirmed that there are strong associations between weather factors and the occurrence of crop pests and diseases. Then we proposed an LSTM network-based method to predict the occurrence of diseases and insect pests in cotton. Results showed that our LSTM-based model outperformed other traditional prediction models.

## Methods

**Fig. 1:**
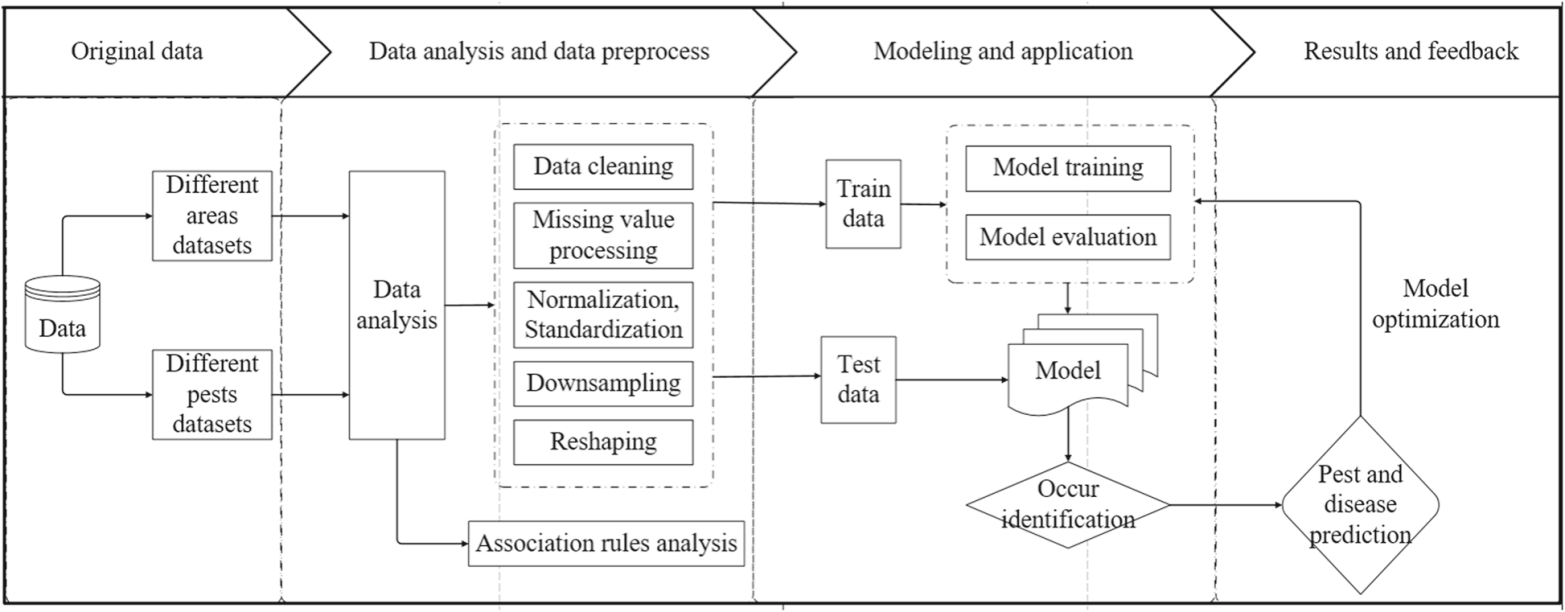
Overview of analysis and prediction of cotton pests and diseases

The flowchart of the whole work is show in Fig. 1. First, the selected datasets in different areas and different pests were analyzed and preprocessed by association rules and data preprocess. Second, the preprocessed data was divided into training dataset and test dataset for building and testing the model of pest occurrence prediction. Finally, results were achieved after optimizing the prediction model.

### Material and Dataset

**Fig. 2:**
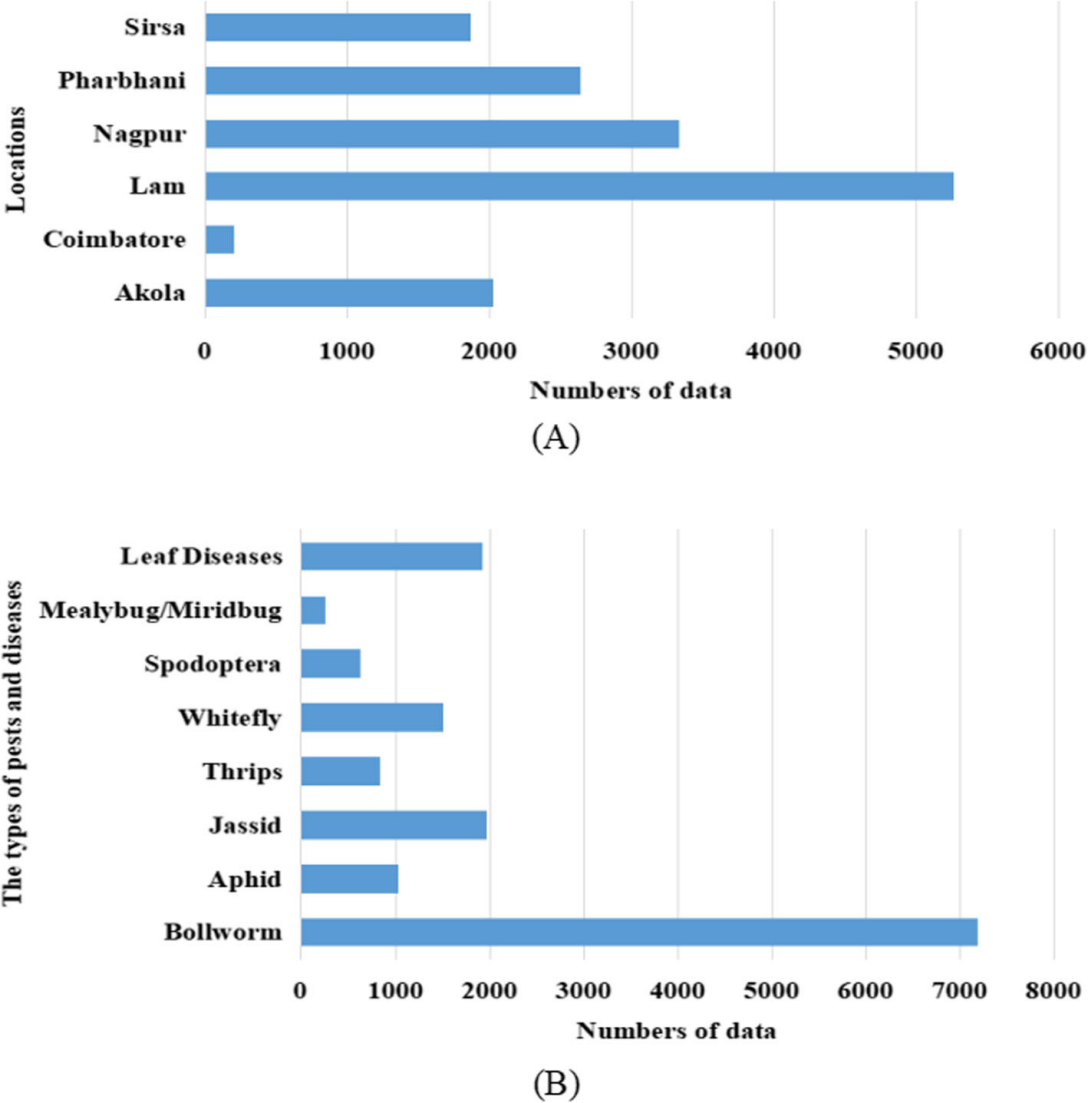
Classification and statistics of cotton pests and diseases in India. **a** Cotton pests and diseases in different regions of India. **b** The occurrence of different types of cotton diseases and insect pests in India

Occurrence of cotton pests and diseases is related by a number of weather factors, however, the interactions among these factors are very complicated. Here, cotton pests and diseases datasets from Crop Pest Decision Support System (http://www.crida.in:8080/naip/AccessData.jsp) were used, where cotton documents was recorded weekly (*15,343*) and contained 10 insect pests and diseases in cotton along with corresponding weather conditions, across 6 important regions in India. Several time series of weather features are applied in the occurrence of pests, including Maximum Temperature *MaxT* ^∘^C, Minimum Temperature *MinT* ^∘^C, Relative Humidity in the morning (*RH1* (*%*)), Relative Humidity in the evening (*RH2* (*%*)), Rainfall (*RF* (*mm*)), Wind Speed (*WS* (*kmph*)), Sunshine Hour (*SSH* (*hrs*)) and Evaporation (*EVP* (*mm*)). The historical records were used to predict future occurrence of pests and diseases. A total of 63 datasets of cotton pests and diseases are obtained from the website. Figure 2a and b provide simple statistics on different types and locations of cotton pests and diseases, respectively.

It can be clearly seen from Fig. 2b that the Bollworm is an important pest infestation in cotton boll stage. It is widely distributed in the world and mainly eats buds, flowers, bells and young leaves, which caused great economic loss for crops such as cotton and that is the main target of biological control. Therefore, we tried to use cotton bollworm records to build weather-pest forecasting model.

### Association rules analysis

Applications of the association rule have been made across multidisciplinary fields, including Web usage mining, intrusion detection, continuous production, and bioinformatics, etc [20, 21]. The goal of association rules mining is to establish the relationship between a set of input variables and a set of output variables [22]. Two important indices for an association rule are support and confidence.

In this paper, we analyzed the association rules of cotton pest and disease records. Because the input of the Apriori algorithm must be discretized data, here the K-means clustering method was adopted to discretize different weather factors, and then the Apriori algorithm was used to mine association rules for the discretized data. Finally, the matched association rules are selected, based on the minimum support and minimum confidence set. Then only the rules that lead to the occurrence of cotton pests and diseases are considered.

### K-means clustering

**Fig. 3:**
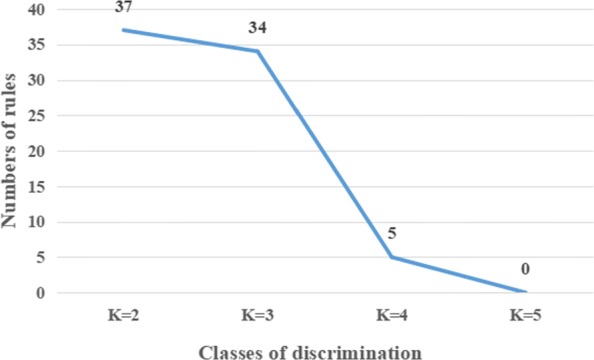
Numbers of association rules directly related to the occurrence of pests and diseases under different K values

k-means clustering is a method of vector quantization, which is popular for cluster analysis in data mining. In order to divide the weather data into more categories, more association rules related to pest occurrence need to be retained. Therefore, all the pest data (*15,343* records) were used to select the *K* value of the k-means clustering. Here, let’s set *minisupport* = 0.05 and *miniconfidence* = 0.5. Figure 3 shows the number of rules that directly lead to the occurrence of pests and diseases under different *K* values. When *k* = 3, more association rules can be obtained, and the data can be better discretized. Table 1 lists the range of intervals after eight weather factors have been discretized. In order to facilitate the records in the association rule analysis, A, B, C, D, E, F, G, H and P to represent MaxT(^∘^C), MinT (^∘^C), RH1 (%), RH2 (%), RF (mm), WS (kmph), SSH (hrs), EVP (mm) and pest occurrence were adopted.

**Table 1:** Coefficient range for different weather factors

Weather features	Coefficient range of each features		
A-*MaxT(∘ C)*	A1 (0, 27.79]	**A2 (27.79, 35.63]**	A3 (35.63, 46.60]
B-*MinT(∘ C)*	B1 (0, 13.68]	B2 (13.68, 21.05]	**B3 (21.05, 32.20]**
C-*RH1(%)*	C1 (0, 57.50]	C2 (57.50, 78.58]	**C3 (78.58, 97.30]**
D-*RH2(%)*	D1 (0, 36.37]	D2 (36.37, 57.89]	**D3 (57.89, 90.40]**
E-*RF(mm)*	**E1 (0, 29.12]**	E2 (29.12,103.52]	E3 (103.52, 602.00]
F-*WS(kmph)*	**F1 (0, 5.73]**	F2 (5.73, 25.52]	F3 (25.52, 71.40]
G-*SSH(hrs)*	G1 (0, 4.97]	**G2 (4.97, 8.03]**	G3 (35.63, 12.70]
H-*EVP(mm)*	**H1 (0, 10.16]**	H2 (10.16, 23.09]	H3 (23.09, 72.00]

Bold means that the weather condition is more likely to cause cotton pests and diseases to occur according to the mined association rules

### Problem formulation

This work aims to predict the occurrences of cotton pests and diseases. Suppose that *X* is the vector set of weather feature records, and *Y* denotes the occurrence of cotton pests and diseases. Giving the training feature vectors $\left (X^{i}_{t0_{i}},Y^{i}_{t0_{i}}\right)$, *i*=1...*N*, our aim is to build a model to capture the relationship among *X* ^*i*^ and *Y* ^*i*^, and therefore to identify the occurrence ($Y^{j}_{t1_{j}}$=1, *j*=1...*M*) or non-occurrence ($Y^{j}_{t1_{j}}$=0, *j*=1...*M*) of cotton pests and diseases for the future test vectors $X^{j}_{t1_{j}}$, *j*=1...*M*, where time *t*0 are earlier than time *t*1. So the prediction problem can be formulated as a binary classification problem, according to the past weather factors (*X*) and pest values (*Y*).

### Long short term memory

LSTM has an end-to-end working mode like neural network, which automatically processes input data and gets people’s desired results [23]. It does not require complex feature selection and model testing as traditional machine learning. Once LSTM network training was completed, it only need to update network parameters based on new data, without building the model again. In recent years, researchers have improved the structure of LSTM, such as Gated Recurrent Unit (GRU) [24] and bidirectional LSTM (*Bi*-LSTM) [25], making it more applicable and more efficient in prediction performance and training time.

**Fig. 4:**
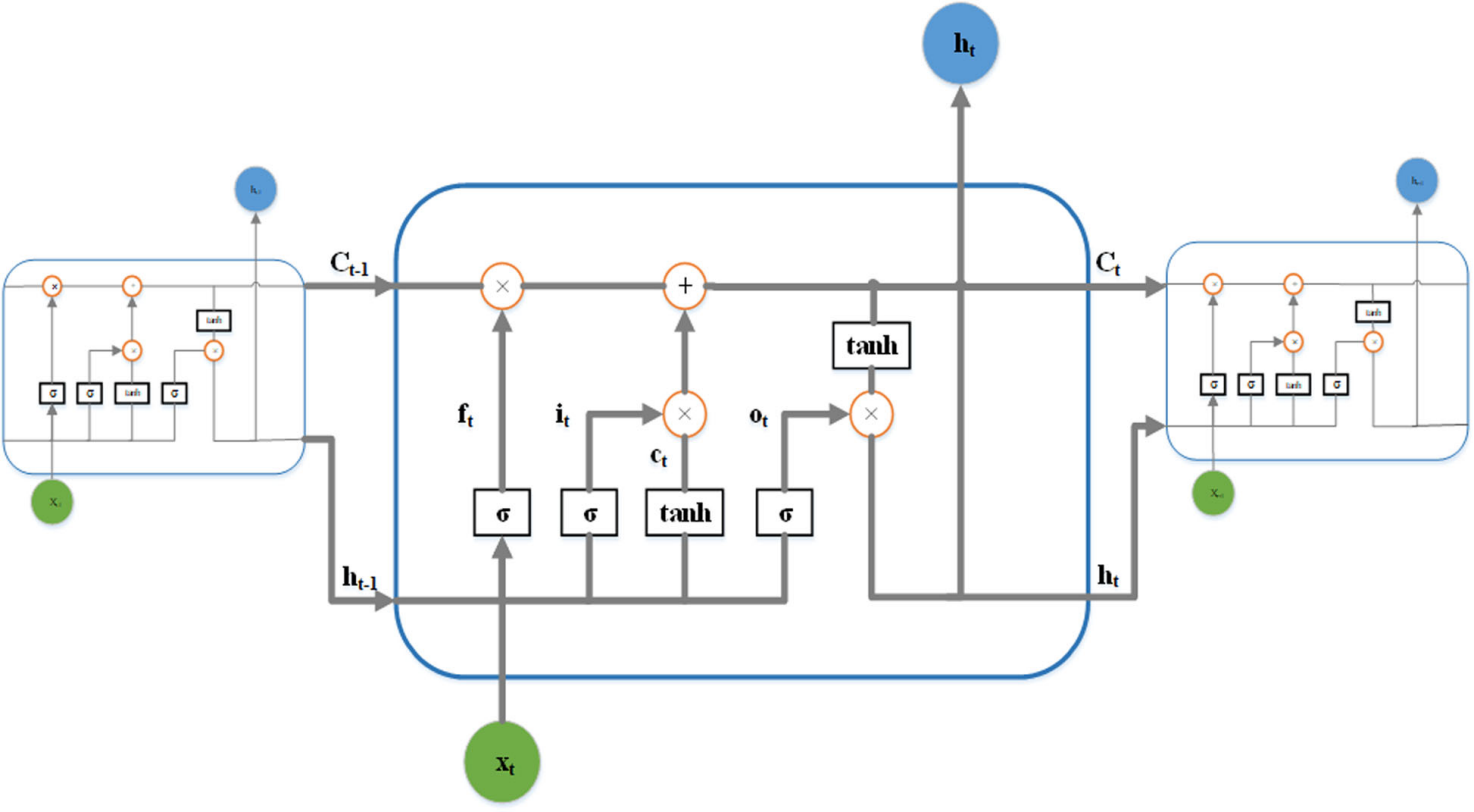
Structure of LSTM cells

LSTM was adopted to capture potential relationship among weather-pest time series data. There are three doors in LSTM. The input gate decides the input *x*_*i*_ entering into the current cell, the forget gate decides if and how much information is forgot for the previous memory, and the output one controls the information outputting from the current cell. The gating operation ultimately determines which information is forgot and which information enters into the neural network as useful information. For the weather-pest forecasting issue, it processes a series of temporal dependency inputs *x*_t_ at time *t* and the hidden vector *h*_*t*−1_from the last time step then gets the predicted *h*_*t*_. The basic structure of LSTM cells can be seen in Fig. 4.

1 $$ \begin{aligned} &{i_{t}} = \sigma \left({{W^{i}} \cdot [{h_{t - 1}},{x_{t}}] + {b^{i}}} \right)\\ &{f_{t}} = \sigma \left({{W^{f}} \cdot [{h_{t - 1}},{x_{t}}] + {b^{f}}} \right)\\ &{o_{t}} = \sigma \left({{W^{o}} \cdot [{h_{t - 1}},{x_{t}}] + {b^{o}}} \right)\\ &{{\mathrm{c}}_{t}} = \tanh \left({{W^{c}} \cdot [{h_{t - 1}},{x_{t}}] + {b^{c}}} \right)\\ &{C_{t}} = {f_{t}} \cdot {C_{t - 1}} + {i_{t}} \cdot {c_{t}}\\ &{{\mathrm{h}}_{t}} = {o_{t}} \cdot \tanh \left({{C_{t}}} \right),\\ \end{aligned}  $$

where *σ* is the sigmoid function; tanh(*) is a nonlinear activation function; *W* is the recurrent weight matrix; *b* is the corresponding bias vector; *i*, *f* and *o* are the outputs of the input, forget, and output gates, respectively; and *C* and *h* are the memory vector and out vector of the cell, respectively.

According to the previous work [26], the output, (*h*_*t*_, *C*_*t*_), of a cell can be represented as a whole function *LSTM*(*):

2 $$ \begin{aligned} \left({{h_{t}},{C_{t}}} \right) = LSTM\left({\left[ {{h_{t - 1}},{x_{t}}} \right],{C_{t-1}},W} \right), \end{aligned}  $$

where *W* concatenates the four weight matrices *W* ^*i*^, *W* ^*f*^, *W* ^*o*^ and *W* ^*c*^.

### Architecture of the LSTM network

The developed model mainly have two parts, the LSTM layers and fully connection layers. The former can capture the temporal relationship between weather data and the occurrences of pests and diseases.The latter can reduce the dimensionality of outputs and then map the reduced output vector to a final prediction.

To implement LSTM, the weather-pest time series data should be converted to 3D tenser (*N*_*samp*_, timesteps, *N*_*feat*_). Here, *N*_*samp*_ is set as the number of samples, timesteps as 4 and *N*_*feat*_ as 9 including eight weather features and one pest values. The final prediction can be defined as below:

3 $$ \begin{aligned} &({h_{i}},{C_{i}}) = LSTM([h_{i - 1},{x_{i}}], C_{i-1},W)\\ &prediction = \sigma (W^{fc} \cdot {h_{l}} + b^{fc}),\\ \end{aligned}  $$

where (*h*_*i*_, *C*_*i*_) denotes the output of the *i*-th cell of LSTM; *σ* is the sigmoid function; *h *_*l*_ is the hidden vector in the last time step of LSTM layer; *W* ^*fc*^ and *b *^*fc*^ are the weight matrix and bias term in full-connection layer, respectively; *prediction* = {0, 1} is the classification result of LSTM network.

To identify whether pests and diseases will occur in the future. We should determine how long the historical observations should be used to the prediction. Of course the longer the historical data is, the better the prediction will be, however the more computation it will need. Here the ‘*timesteps*’ is set as 4, i.e., four samples of weather-pest data are input together into the LSTM. In addition, three parameters for the whole structure of the network should be determined: the number of layers for LSTM layer *l*_*r*_, the full-connected layer *l*_*fc*_ and the corresponding number of hidden units denoted by *units_r*.

In addition, to train the network, some critical parameters have to be determined, such as the optimization method, the learning rate, the batch size, etc. Stochastic gradient descent (SGD) is a standard algorithm for training artificial neural networks [27], The details of gradient descent and the parameters of network can be seen in Eq. (4):

4 $$ \begin{aligned} &{g_{t}} = \frac{{d{f_{t}}\left(\theta \right)}}{{d\theta }}\\ &\theta_{t} = \theta_{t - 1} - \eta \cdot {g_{t}},\\ \end{aligned}  $$

where *f*_*t*_(*θ*) is the objective function used in the LSTM network; *η* is the learning rate; *θ* is parameter vector of network.

Here, binary-crossentropy was adopted as the loss function of the binary classification, whose definition is shown in below,

5 $$ {f_{t}}\left(\theta \right) = - \sum y_{t}^{true} \cdot \log \left({y_{t}^{prediction}} \right),  $$

where $y_{t}^{true}$ is the actual value; $y_{t}^{prediction}$ is the prediction value of network, which is calculated by Eq. (4).

Moreover, it is difficult for SGD optimizer to find a best learning rate for non-stationary objectives, therefore it usually falls into a local optimal solution. RMSProp (for Root Mean Square Propagation) [28] was adopted instead of SGD to optimize our model, which has shown excellent adaptation of learning rate in different applications. The idea is to divide the learning rate for a weight by arunning average of the magnitudes of recent gradients for that weight. The relevant formula of the algorithm is as follows:

6 $$ v_{t} = v_{t - 1} \cdot \gamma + (1 - \gamma) \cdot {g_{t}}^{2} \theta_{t} = \theta_{t - 1} - \frac{\eta }{\sqrt {v_{t}}} \cdot {g_{t}},  $$

where *v* is the raw moment estimate; *γ* is the forgetting factor; *η* is the learning rate; *θ* is parameter vector of network.

### Performance measurement

Accuracy (*ACC*) [29], Area Under the Curve (*AUC*) [30] and *F1-score* were used to measure the effectiveness of prediction methods. Each binary classification model outputs only two types, positive class and negative class (records as *P* and *N*). Therefore bivariate model has four outcomes for the case predictions: true positive (*TP*), true negative (*TN*), false positive (*FP*), and false negative (*FN*). The definitions of *ACC* and *F1-score* are shown in below:

7 $$  \begin{aligned} &ACC = \frac{{TP + TN}}{{P + N}}\\ &F1{\mathrm{ - score}} = \frac{{2TP}}{{P + P'}}.\\ \end{aligned}  $$

In addition, Receiver Operating Characteristic (*ROC*) curve was introduced and the area under the ROC curve (*AUC*) can be used to evaluate a classifier. The definitions of *AUC* is shown as below:

8 $$ AUC = \frac{{\sum\nolimits}_{i \in positiveClass} {rank_{i}- \frac{M(M+ 1)}{2}} }{M \times N},  $$

where *M* and *N* are the numbers of positive class and negative class, respectively; we sort the probability values of each sample predicted by the model from small to large, and *rank*_*i*_ represents the serial number of the *i*-th sample. *i*=1,...,*n*; *n*is the number of total data, *n* = *M*+ *N*.

### Implementation

Other traditional classification models, i.e., support vector machine (SVM), k-NearestNeighbor (KNN) and random forest, were also implemented for cotton pests occurrence prediction for comparing with our LSTM model. The experiments were run under the environment of Intel (R) Core (TM) i7-4790 CPU @3.60GHz (8CPUs), 8G RAM, Windows 10 64 bits operating system, programmed with Python 3.6. The proposed network was implemented with TensorFlow 0.11 [31], while SVM was implemented by Scikit-learn [32].

## Experiment and results

### Association rules analysis

The used Apriori algorithm is mainly divided into two steps, finding frequent itemsets and generating association rules.

First, let *I*=*i*_1_,...,*i*_*k*_ be a set of *k* items. A basket dataset *B*=(*b*_1_,...,*b*_*n*_) is any collection of *n* subsets of *I* and each subset *b*_*i*_⊆*I* is called a basket of items. We suppose that two sets of items, *A* and *B*, do not intersect. Given support and confidence, there is an established association rule: *A* (called antecedent) →*B* (called consequent). This rule satisfies the following conditions: (a) *A* and *B* occur together in at least *support*×100*%* of the *n* baskets; (b) Among those baskets containing A, at least *confidence*×100*%* also contain *B*. Then, the significance of an association relationship between A and B can be measured by the support and the confidence,

9 $$  \begin{aligned} &S{\text{upport}} (A \to B) = P(A \cup B) = \frac{nAB}{n}\\ &Confidence(A \to B) = P(B|A) =\frac{nAB}{nA}, \\ \end{aligned}  $$

where *A*→*B* represents an association rule between A and B; *n* is the total number of items in the population; *nA* is the total number of items in A; and *nAB* is the total number of items in both A and B.

Apriori algorithm filters frequent items based on minimum support. Then, a connection rule is established between frequent items, and the confidence of the connection rule is calculated, and the association rule satisfying the minimum confidence can be finally retained. Currently, the thresholds of the support and the confidence are set arbitrarily by users and it is very difficult to interpret the result. If the thresholds of the support and the confidence are set too low, many rules will be established. On the other hand, if the thresholds are set too high, no rules may be established [33]. After many attempts, we found that there have almost no association rule information under higher thresholds. One of the reasons may be that different datasets (i.e., different types of pest and disease datasets) have their own specific association rules. When we put all datasets together, the disappearance of these specific association rules leads to a decrease in the number of association rules generated by the dataset as a whole. However, in order to make the results universal, we need to perform the same pre-processing on all data sets, that is, discretization in the same way, which requires all the samples to be processed together. Therefore, we set a lower threshold with a minimum support of 0.05 and a minimum confidence of 0.5. Any association rule must meat these minimal support and minimal confidence values to be meaningful.

After discretization based on the method in Table 1, we analyzed the association rules for the different locations of pest and disease datasets and the different types of pest and disease datasets as show in Fig. 2a and b, respectively. Some of the results are shown in Tables 2 and 3. The tables list the number of association rules associated with the occurrence of cotton pests and diseases and the top three association rules (If the number of rules included in the top one or top two confidences exceeds three, we will not show them all here.) under the minimum support and minimum confidence. For example, the rule: *A*2,*B*3,*D*3,*F*1→*P*,*support*=0.0927,*confidence*=0.8592, indicating that when the Maximum Temperature in (27.79, 35.63], the Minimum Temperature in (21.05, 32.20], Relative Humidity in the evening in (57.89, 90.40], and Wind Speed in (0, 5.73], the probability of occurrence of pests and diseases is 85.92%, and the probability of this occurrence is 9.27%.

**Table 2:** Partial association rules between pest occurrence and weather factors in five different regions (25 rules)

Locations	Numbers	Association rules of pests occur and weather factors
Akola	241	*B2,C3,E1,H1 →P, A2,B2,C3,E1 →P,B2,C3,E1,F1 →P, B2,C3,E1 →P,*
		*A2,B2,C3,E1,F1 →P, A2,B2,C3,E1,H1 →P,B2,C3,E1,F1,H1 →P, A2,B2,C3,E1,F1,H1 →P,*
		Support= 0.07643, confidence=0.790816
Lam	94	*A2,B3,D3,F1 →P*, Support=0.0873694, confidence=0.569307;
		*A2,D3,E1,F1 →P*, Support=0.0907882, confidence=0.567023;
		*A2,B3,C3,D3,F1 →P*, Support=0.0849003, confidence=0.565823;
Nagpur	80	*A2,C3,E1,G2 →P*, *A2,C3,E1,F1,G2 →P*, Support=0.0615986, confidence=0.638629;
		*A2,E1,H2 →P*, *A2,E1,F1,H2 →P*, Support=0.0585938, confidence=0.621019.
Pharbhani	44	*A2,D2,F1 →P*, *A2,D2,F1,H1 →P*, Support=0.0631619, confidence=0.594306;
		*A2,C3,D2,E1 →P*, *A2,C3,D2,E1,H1 →P*, Support=0.0540847, confidence=0.581301;
Sirsa	121	*A2,B3,D3,E1 →P*, *A2,B3,D3,E1,F1 →P*, Support=0.0502674, confidence=0.87037;
		*B3,D3,E1 →P*, *B3,D3,E1,F1 →P*, Support=0.0572193, confidence=0.856;

**Table 3:** Partial association rules between pest occurrence and weather factors in five different types of cotton pests and diseases (15 rules)

Pests and Diseases	Numbers	Association rules of pests occur and weather factors
Ahpid	153	*A2,E1,H3 →P,* Support = 0.0523, confidence =0.8438;
		*A2,F1,H3 →P,* Support = 0.0630, confidence =0.7927;
		*A2,C3,F1,H3 →P,* Support = 0.0620, confidence =0.7902;
Jassid	199	*B3,D3,F1,H1 →P*,Support =0.0527, confidence =0.8814;
		*A2,B3,D3,F1 →P*,Support =0.0927, confidence =0.8592;
		*A2,B3,F1,G2 →P*,Support =0.0567, confidence =0.8550;
Leaf Diseases	142	*A2,D3,E1,F1,G2 →P*, Support =0.0577, confidence =0.7762;
		*A2,C3,D3,E1,F1,G2 →P*, Support =0.0541, confidence =0.7704;
		*A2,D3,E1,F1 →P*, Support =0.0873, confidence =0.7534.
Thrios	109	*A2,C3,G2,H1 →P*, Support =0.0709, confidence =0.7024;
		*A2,B3,F1,H1 →P*, Support =0.0505, confidence =0.7000;
		*A2,B3,C3,E1,H1 →P*, Support =0.0637, confidence =0.6883.
Whitrfly	52	*A2,B3,C3,F1 →P*, Support =0.0729, confidence =0.6667;
		*A2,B3,F1 →P*, Support =0.0816, confidence =0.6613;
		*A2,B3,D3,F1 →P*, Support =0.0657, confidence =0.6600.

**Fig. 5:**
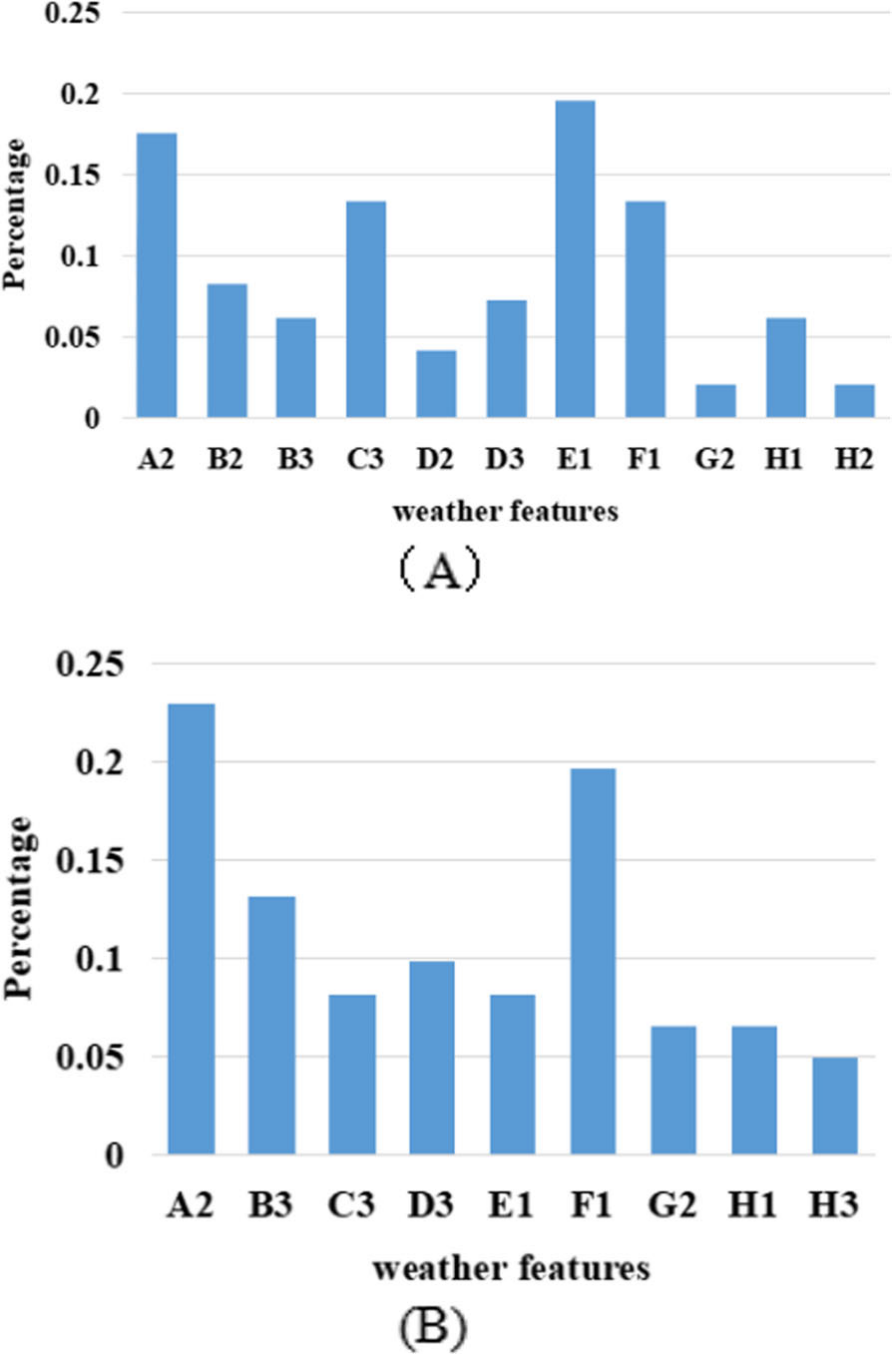
The probability of occurrence of each item in the association rules. **a** five different regions; **b** five different types of cotton pests and diseases.

In order to further analyze the impact of weather factors on the occurrence of cotton pests and diseases, we separately counted the left items of all the rules (25 rules and 15 rules) listed in Tables 2 and 3. then, we simply calculate the probability of each item. The results are shown in Fig. 5 It can be seen from the figure that the factors affecting the occurrence of cotton pests and diseases are concentrated, i.e., suitable temperature, humid air, low rainfall and wind speed, which are more likely to cause pests and diseases. In addition, there are some differences in the two subfigures Fig. 5a and b, i.e., Fig. 5a shows that the factors affecting cotton pests and diseases in different regions are more extensive, while Fig. 5b concentrated more. Perhaps cotton grown in different areas is affected by more complex factors during its growth.

### Determination of parameters

There are a total of 63 time series datasets in the Crop Pest Decision Support System, in which the sizes of the datasets range from 52 to 1196. In order to guarantee the accuracy and generalization ability of the network, we use the top size datasets to train our model. Five datasets of bollworm records with top size, which are denoted as p1, p2, p3, p4 and p5, are selected to train the LSTM network and determine the network parameters. Table 4 shows the size of five groups of cotton bollworm records.

**Table 4:** The size of five groups of cotton bollworm records

	P1	P2	P3	P4	P5
Pests and diseases	335	316	167	197	70
No pest and disease	861	724	457	286	190
Total	1196	1040	624	483	260

Before training the LSTM model, each dataset is divided into a training set and a test set, where the first three quarters of the dataset is regarded as the training set and the rest records as the test set. Firstly, we fix *l*_*r*_ and*l*_*fc*_ as 1, and choose a proper value of *units_r* from {4, 5, 6, 7}. Table 5 shows the predictions on five datasets of bollworm with different values of *units_r*.The boldface items in the table represent the best performance, i.e. the largest *ACC*, *AUC* and *F1-score*. It can be seen from the results that the best performance occurs when *units_r* = 5 on three datasets p1, p2 and p4. Although the model performs not good enough when *units_r* = 7 on dataset p3 and *units_r* = 6 on dataset p5, it can be seen that the performance difference of the model and those with other *units_r* is not obvious. In addition, there are fewer positive samples in the P3 and P5 datasets, which may also be one of the reasons for the difference in network predictions. So in the following experiments, we set *units_r* as 5.

**Table 5:** Predictions on five datasets in terms of *units_r*

Units_r	Metrics	P1	P2	P3	P4	P5
4	ACC	0.9241	0.8973	0.9111	0.9017	0.8742
	AUC	0.9712	0.9532	0.9687	0.9578	0.9465
	F1-score	0.8857	0.8258	0.8316	0.8737	0.7804
5	ACC	**0.9329**	**0.9169**	0.9176	**0.9136**	0.8903
	AUC	**0.9764**	**0.9674**	0.9663	**0.9704**	**0.9715**
	F1-score	**0.8949**	**0.8555**	0.8580	**0.8955**	0.7903
6	ACC	0.9281	0.9063	0.9098	0.8949	0.8968
	AUC	0.9737	0.9643	0.9529	0.9628	0.9649
	F1-score	0.8896	0.8450	0.8420	0.8680	**0.8234**
7	ACC	0.9276	0.9013	**0.9255**	0.9000	**0.9032**
	AUC	0.9710	0.9557	**0.9717**	0.9551	0.9636
	F1-score	0.8870	0.8205	**0.8584**	0.8763	0.8104

The entry in boldface represents the best performance on one dataset with respect of Units_r

Then, we also use the time series sequences from the same five groups data in order to choose a proper value for *l*_*r*_ from {1,2,3}, the other two parameters are set by *units_r*= 5 and *l*_*fc*_= 1. Table 6 shows the results on five datasets with different values of *l*_*r*_. The boldface items in the table represent the best performance for each dataset. Results shows that the best performance occurs when *l*_*r*_= 1. The reason may be due to the increasing number of weights with increasing recurrent LSTM layers, which results in insufficient dataset to train larger amount of weights. Actually, experiences show that LSTM with more layers did not always perform good. Results in this work show that more LSTM layers yield unstable results more likely. Therefore in the following experiments, we set *l*_*r*_ as 1.

**Table 6:** Prediction results on five datasets in terms of *l*_*r*_

*l*_*r*_	Metrics	P1	P2	P3	P4	P5
1	ACC	**0.9354**	**0.9041**	**0.9216**	**0.8983**	**0.9161**
	AUC	**0.9774**	**0.9595**	**0.9748**	**0.9622**	0.9604
	F1-score	0.8918	**0.8183**	**0.8633**	**0.8817**	0.8290
2	ACC	0.9331	0.8831	0.9190	0.8949	0.8936
	AUC	0.9727	0.9402	0.9656	0.9567	0.9657
	F1-score	**0.8931**	0.7844	0.8374	0.8710	0.7972
3	ACC	0.9245	0.8784	**0.9216**	0.8847	0.9129
	AUC	0.9732	0.9377	0.9598	0.9421	**0.9712**
	F1-score	0.8769	0.7806	0.8559	0.8564	**0.8445**

The entry in boldface represents the best performance on one dataset with respect of lr

Lastly, we still use the same five groups datasets to choose a proper value for *l*_*fc*_ from {1, 2, 3} and its units. Table 7 shows the results with different values of *l*_*fc*_. The numbers in the square brackets stand for the number of the hidden units. The boldface items in the table represent the best performance for each dataset. The model achieves the best performance when *l*_*fc*_ = 2. The reason is similar to that in the choose of *l*_*r*_, i.e., the model with more layers means there are more weights to be trained and more computation it needs. So in the following experiments, we set *l*_*fc*_ = 2, while the numbers of the hidden units are 5 and 1. The final full connectivity layer is integrated into the LSTM model to yield the predictions of pests and diseases.

**Table 7:** Prediction results on five datasets in terms of *l*_*fc*_

*l*_*fc*_	Metrics	P1	P2	P3	P4	P5
1[2]	ACC	0.9300	0.8858	0.9189	0.8780	0.8936
	AUC	**0.9735**	0.9515	0.9668	0.9565	0.9510
	F1-score	0.8819	0.8080	0.8545	0.8378	0.8256
2[5,1]	ACC	0.9206	**0.9020**	**0.9320**	**0.8865**	**0.9129**
	AUC	0.9694	**0.9626**	**0.9738**	**0.9517**	**0.9640**
	F1-score	0.8662	**0.8285**	**0.8735**	**0.8616**	**0.8660**
3[5,5,1]	ACC	0.9292	0.8959	0.9124	0.8729	0.8903
	AUC	**0.9695**	0.9512	0.9506	0.9381	0.9555
	F1-score	0.8859	0.8264	0.8466	0.8550	0.8237
3[10,5,1]	ACC	0.9284	0.8946	0.9020	0.8763	0.9129
AUC	**0.9695**	0.9529	0.9443	0.9526	0.9625	
	F1-score	**0.8877**	0.8288	0.8327	0.8480	0.8363

The entry in boldface represents the best performance on one dataset with respect of lfc

### Performance of LSTM

**Fig. 6:**
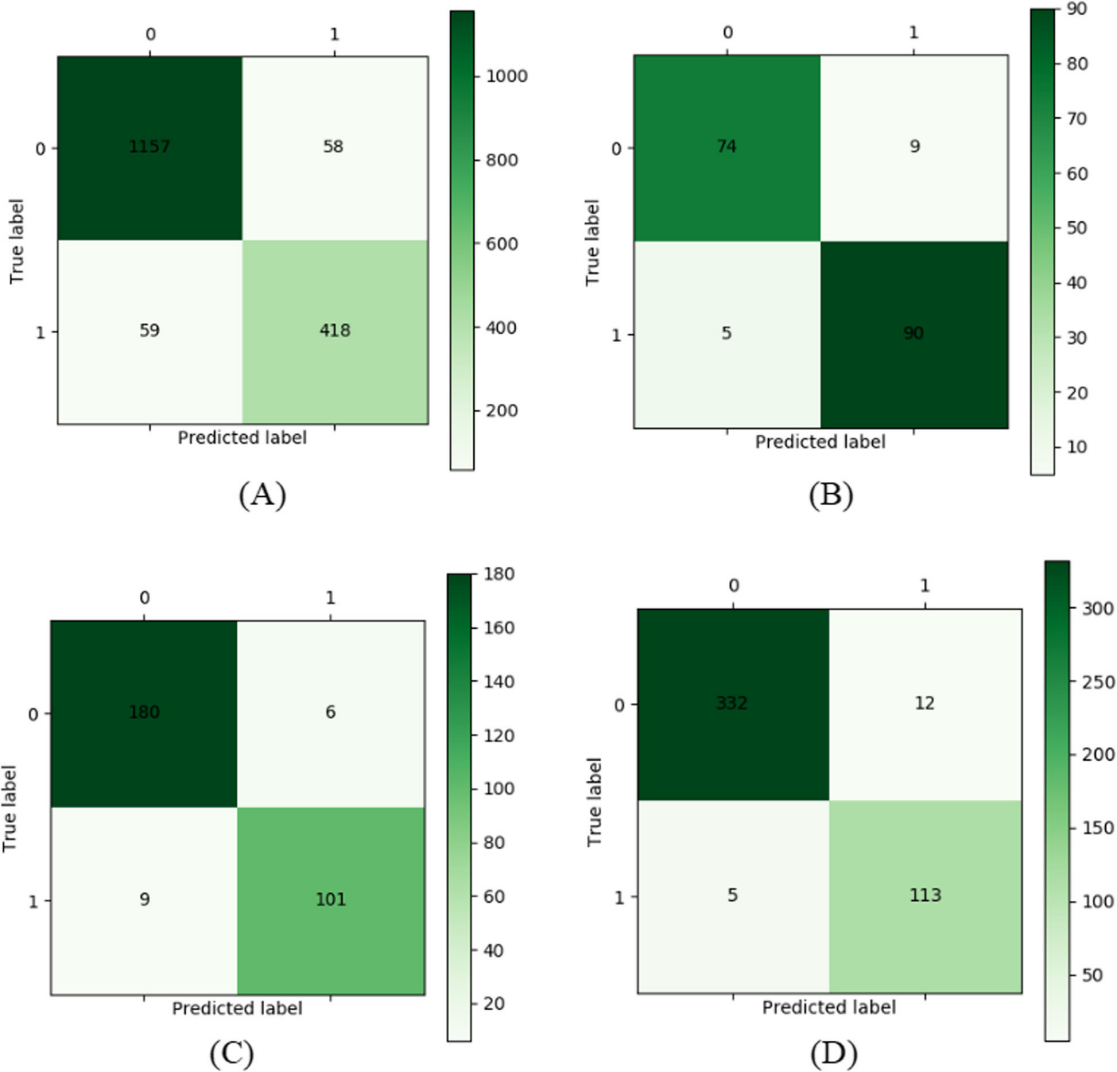
Confusion matrix on the four kinds of dataset with LSTM network. Subfigures A, B, C and D show the confusion matrix of cotton pests and diseases occurrence of bollworm, whitefly, jassid and leaf blight, respectively. Here, the green bar representative model predicts the correct number of samples. **a** Bollworm **b** Whitefly **c** Jassid **d** Leaf blight

**Fig. 7:**
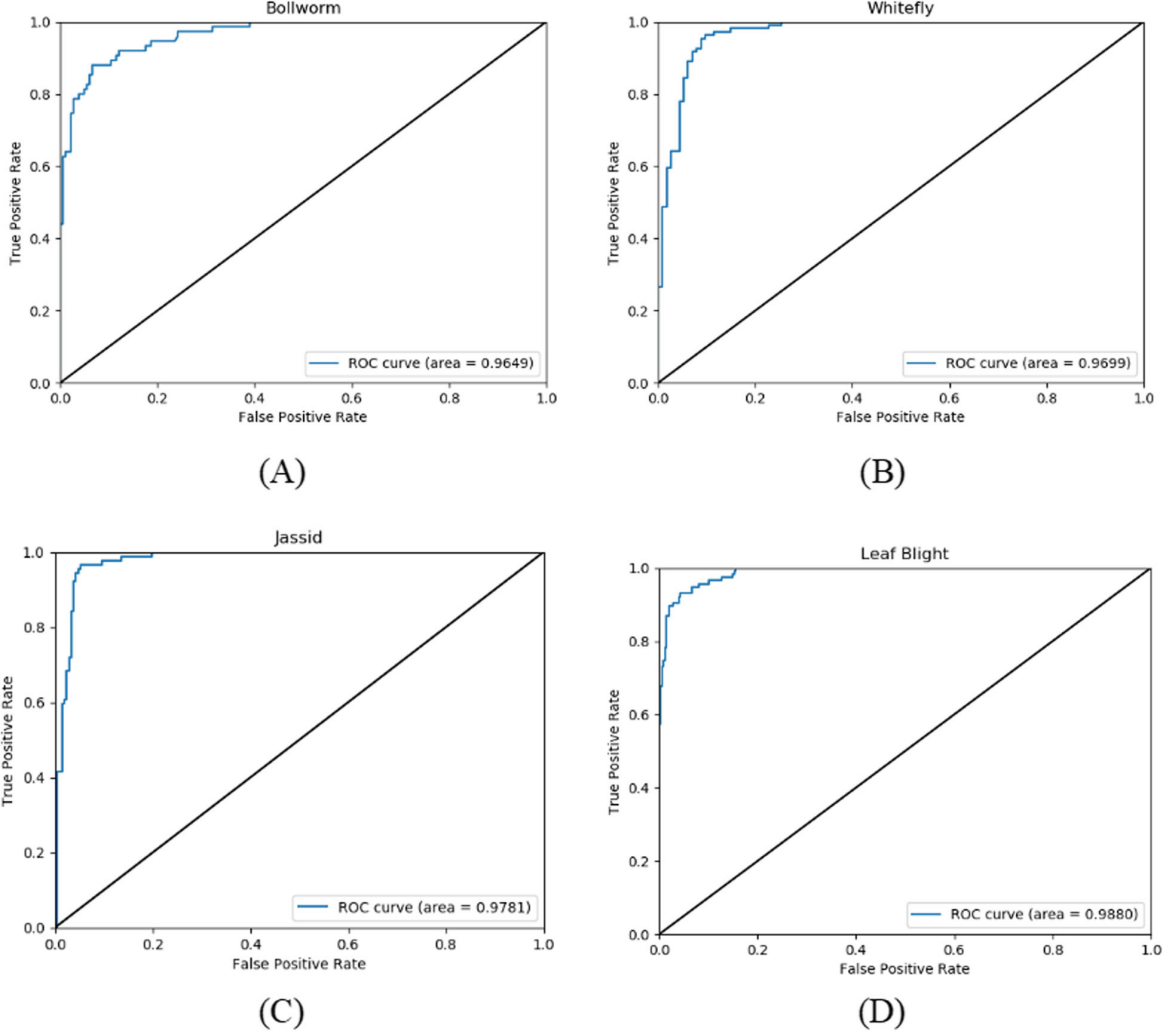
ROC curves on the four kinds of dataset with LSTM network. Subfigures 7A, 7B, 7C and 7D show the ROC curves of cotton pests and diseases occurrence of bollworm, whitefly, jassid and leaf blight, respectively. Here "area" means the area under each ROC curve. **a** Bollworm **b** Whitefly **c** Jassid **d** Leaf blight

As discussed in above, the parameters of the used LSTM is listed in Table 8. After building the basic framework of the LSTM network, the other parameters have to be adjusted to expect the model achieving higher performance, i.e., *dropout* = 0.1. Compared with traditional machine learning methods, LSTM network can directly update network parameters for new data without having to restart feature selection and rebuild networks. It also can update the network parameters in real time according to the current input data, and can be applied to predict the occurrences of other kinds of pests. We hope our model could be applied in different cotton pests and diseases, so other pests and diseases records, such as jassid, whitely, and the common leaf blight of cotton, are input into the model to show its prediction power. The sizes of records are shown in Table 9. The performance comparison on different kinds of datasets with LSTM network is listed in Table 10. Figures 6 and 7 illustrate the confusion matrix and ROC curves on the three kinds of pests (bollworm, whitefly and jassid) datasets and leaf blight dataset, respectively. From the Table 10 and Fig. 6, our model not only performs well in pests prediction, but also in disease, which exhibits good generalization ability. At the same time, Fig. 7 also shows that our model also has a good representation in the accuracy of classification. All the results indicate that the LSTM network is suitable for the prediction of cotton pests and diseases, which also lays a theoretical foundation for practical application in the future.

**Table 8:** The list of parameters for LSTM network and other compared methods

Methods	Parameters
LSTM	*l*_*r*_=1; *l*_*fc*_=2[5,1]; *units_r*=5
SVM	*type*=’LinearSVC’; *C* = 10
KNN	*weights*=’distance’; *n_neighbors*=3; *algorithm*=’ball_tree’; *p* = 2
Random Forest	*n_estimators*=100

**Table 9:** The sizes of datasets for the four kinds of pests and diseases

	Bollworm	Whitefly	Jassid	Leaf Blight
Pests and diseases	1776	450	730	523
No pests and diseases	5307	1059	1244	1401
Total	7083	1509	1974	1924

**Table 10:** Predictions on different kinds of pests and diseases with LSTM network

Metrics	Bollworm	Whitefly	Jassid	Leaf Blight
ACC	0.9207	0.9244	0.9354	0.9557
AUC	0.9659	0.9687	0.9776	0.9868
F1-score	0.8749	0.9243	0.9161	0.9204

### Prediction comparison with other methods

The bollworm dataset **p1** was adopted to implement the prediction comparison of our proposed method with other classical machine learning methods KNN [34], SVM [35] and Random Forest [36]. The models were trained on the training datasets and the optimal prameters were selected for evaluating the models on the test datasets. For LSTM network, the parameters of *units_r*, *l*_*r*_ and *l*_*fc*_ were set as 5, 1 and 2, respectively; for KNN, *weights* = ‘distance’, *n_neighbors* = 3, *algorithm* = ‘ball_tree’ and *p* = 2; for SVM, LinearSVC was adopted and *C* = 10; for Random Forest, *n_estimators* was set as 100. The detailed discussion on these parameters are not shown in this paper. Moreover, the list of parameters for the models is shown in Table 8.

**Fig. 8:**
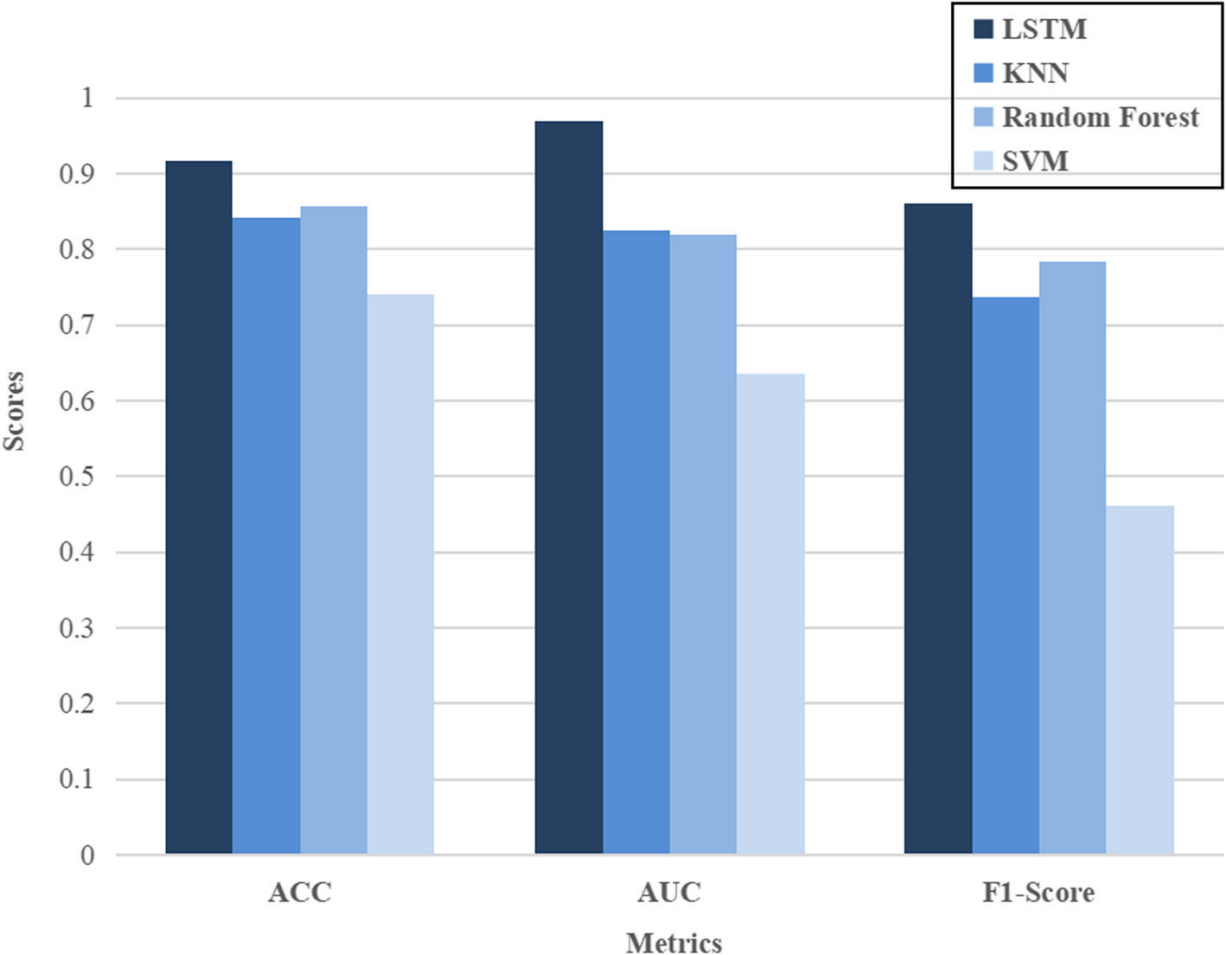
Performance comparison on dataset "p1" with different methods

Figure 8 shows the prediction results. The boldface items in the table represent the best performance, i.e., the largest area average ACC, AUC and F1-score. It can be seen from the results that LSTM network achieves the best prediction performance, KNN and Random Forest are the second, and SVM is the worst. Moreover, the LSTM gets good results, AUC scored 0.97 (two significant figures retained) and ACC achieved 0.92, while, this is difficult to do with traditional machine learning methods. From the results, it may be due to the linear relationship between the weather factors we collected and the occurrence of cotton pests, i.e., in winter, the higher humidity and temperature, the better the overwintering of eggs and the outbreak of pests damage in the coming year, while KNN is superior to nonlinear models such as SVM in dealing with linear problems. However, in addition to a certain linear relationship between weather and pest occurrence, there still have a strong regularity in time. These time rhythm cannot be extracted using feature engineering. RNN has made great breakthroughs in dealing with time series problem, Therefore, the optimal model to solve the problem depends on the internal structure of the problem data, and it is impossible to evaluate the advantages of each model separately.

## Discussion

From this work, there are certain relationship between weather factors and the occurrence of cotton pests and diseases. In autumn and winter, suitable temperature, humidity, low rainfall, low wind speed, suitable sunshine time and low evaporation are more likely to cause cotton pests and diseases (as show in Table 1). Furthermore, the factors affecting cotton pests and diseases in different regions are more extensive, such as warm temperature (A2), low wind speed (F1), and low rainfall (E1). Perhaps cotton grown in different areas is affected by more complex factors during its growth.

The occurrence of cotton pests and diseases is not only related to climatic factors, but also closely related to other factors, i.e., the growth of cotton, the growth cycle and evolution of pests, etc. Moreover, we dropped the pest value feature to train different models, and we found that the law of the occurrence of pests and diseases is also an important feature of model learning, while our model only considering weather factors and historical pest data. Although the proposed model yielded good predictions on different datasets, it seems that it could be greatly improved and it is worth of collecting more effective features to further optimize the network. Furthermore, it is more interesting and meaningful to concern about the pest hazard level of crops in reality. It is a problem of multi-classification and even regression prediction. Therefore, in the future work, we will try to build more datasets with more factor features, including weather factors, the occurrence cycle of pests and diseases and so on. In addition, we will try to use the LSTM network as well as other deep learning methods to predict the hazard level of pests and diseases. It enables people to prevent crop diseases and insect pests in a timely manner.

**Fig. 9:**
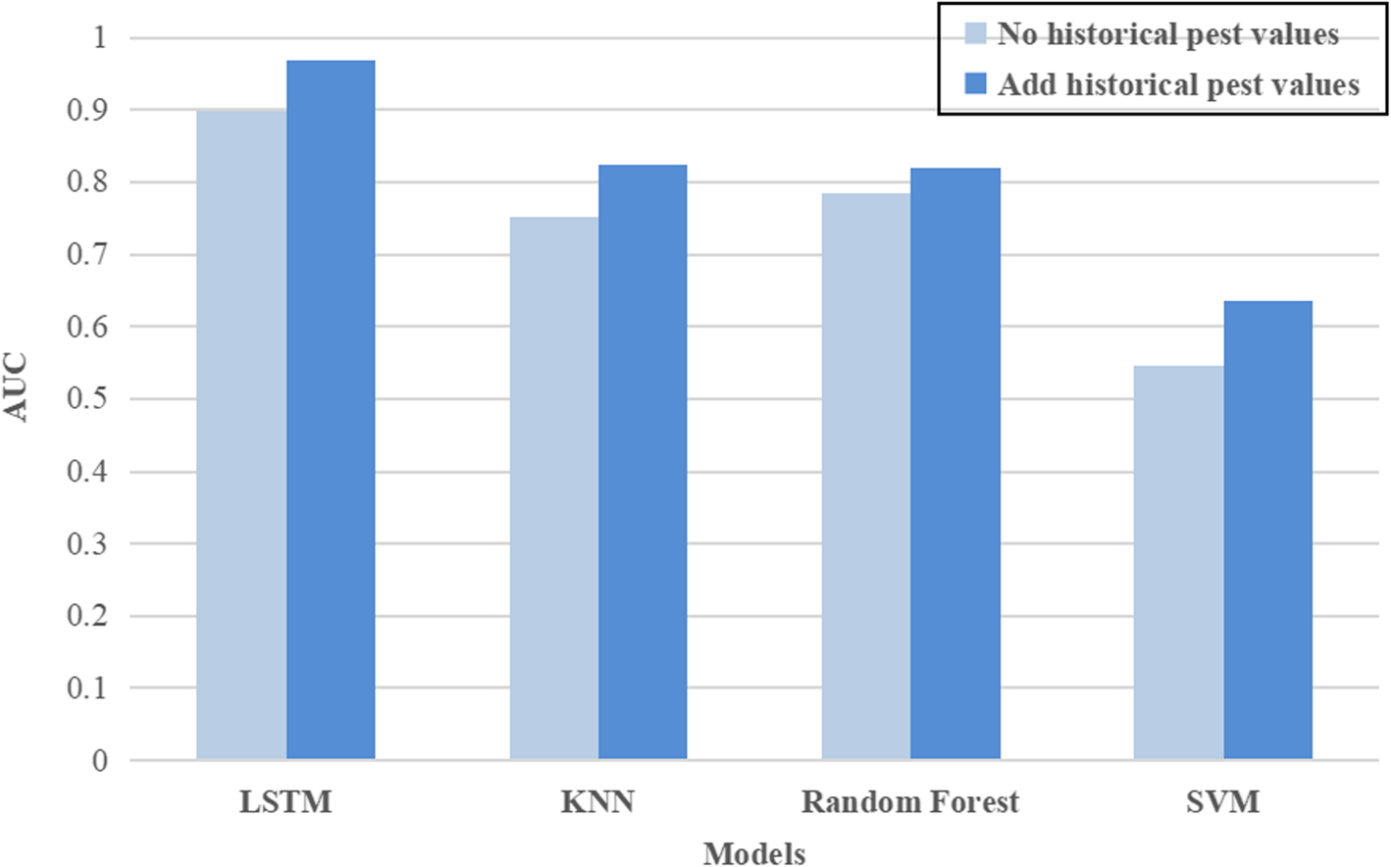
The AUC scores of each model without adding historical data on dataset "p1"

The historical pest values plays an important role in model establishment. Although most of this paper discussed the impact of weather factors on pests and diseases, we cannot ignore the fact that pests and diseases have their own regularity. For example, cotton pests often occur continuously for more than a decade. Based on the existing model, we compared the historical pest values as a feature of model training and the absence of historical data. The results are shown in Tables 10 and 11, respectively. To make the results clearer, we have drawn a bar chart of the AUC scores for the different models as show in Fig. 9. The bar graph shows that models with historical pest and disease data have higher AUC scores. In addition, although the results show that both the machine learning models and the LSTM network perform better after adding historical pest values, the performance improvement of LSTM is more significant, which also re ects the advantages of LSTM in extracting time series information.

**Table 11:** Performance of different models without adding historical pest values on dataset **p1**

Metrics	LSTM	KNN	Random Forest	SVM
ACC	0.8393	0.8135	0.8423	0.7485
AUC	0.8994	0.7515	0.7845	0.5453
F1-score	0.6920	0.6338	0.6861	0.2009

## Conclusions

In this paper, we proposed an LSTM-based classifier that can predict the occurrence of future cotton pests and diseases based on historical data including weather factors and pests data, which is an important thing for the future prevention and control of cotton pests and diseases and the development of agriculture. The neural network is a black box model, it does not need complex feature engineering, and we don’t know which features might be useful for model training. Association rule mining simply counts the weather conditions that affect the occurrence of cotton pests and diseases. Although we do not need to add these features into LSTM network for training in a complex combination, based on these rules, we have more confidence to establish a weather-pest model. This is the first time that we have used LSTM to solve the problem of pest prediction. The proposed model mainly consists of two major parts, the LSTM layers and the fully connected layers. The former is to model the time series data, and the latter is to map the output of LSTM layer to a final prediction.

We explore the optimal setting of this architecture by experiments and report the prediction results of bollworm pests to confirm the effectiveness of the proposed method. In addition, we also investigate the model on different types of cotton pests and diseases records, i.e., jassid, whitrly and leafblight, and achieve good predictions. Moreover, some traditional machine learning methods, i.e., KNN, SVM and Random Forest, are implemented to show the prediction comparison with LSTM model. Results show that LSTM network has certain advantages in processing time-dependent problem, and show the importance of model selection. Although our model outperformed other methods, probably, the features that the datasets contained are insufficient to achieve more accurate predictions.

## Data Availability

Not applicable.

## Abbreviations

ACC: Accuracy
AUC: Area under the curve
Bi-LSTM: Bidirectional LSTM
CNN: Convolutional neural network
dsRNA: Double-stranded RNA
EVP: Evaporation
FN: False negative
FP: False positive
GRNN: General regression neutral networks
GRU: Gated recurrent unit
ICA: Independent component analysis
KNN: k-NearestNeighbor
LSTM: Long short term memory
MaxT: Maximum temperature
MinT: Minimum temperature
MLFN: Multiplier feed-forward neutral networks
NCPHMDA: Network consistency projection for human microbe-disease association prediction
RF: Rainfall
RH1: Relative humidity in the morning
RH2: Relative humidity in the evening
RMSProp: Root mean square propagation
RNN: Recurrent neutral network
ROC: Receiver operating characteristic
SGD: Stochastic gradient descent
SSH: Sunshine hour
SVM: Support vector machine
TN: True negative
TP: True positive
WS: Wind speed
